# Multi-modal imaging and anatomic classification of the white dot syndromes

**DOI:** 10.1186/s40942-017-0069-8

**Published:** 2017-03-20

**Authors:** Meisha L. Raven, Alexander L. Ringeisen, Yoshihiro Yonekawa, Maxwell S. Stem, Lisa J. Faia, Justin L. Gottlieb

**Affiliations:** 1 Department of Ophthalmology and Visual Sciences, University of Wisconsin, 600 Highland Ave, Madison, WI 53705 USA; 2 McPherson Eye Research Institute, Madison, WI USA; 3 Associated Retinal Consultants, William Beaumont Hospital, Royal Oak, MI USA; 4 Department of Ophthalmology and Visual Sciences, University of Wisconsin, 2870 University Ave, Room 206, Madison, WI 53705 USA

**Keywords:** White dot syndromes, Birdshot chorioretinopathy, Multiple evanescent white dot syndrome, Acute posterior multifocal placoid pigment epitheliopathy, Multifocal choroiditis with panuveitis, Serpiginous choroiditis, Relentless placoid chorioretinitis, Punctate inner choroidopathy, Sympathetic ophthalmia, Vogt–Koyanagi–Harada disease

## Abstract

The white dot syndromes (WDS) are a diverse group of posterior uveitidies that share similar clinical findings but are unique from one another. Multimodal imaging has allowed us to better understand the morphology, the activity and age of lesions, and whether there is CNV associated with these different ocular pathologies. The “white dot syndromes” and their uveitic masqueraders can now be anatomically categorized based on lesion localization. The categories include local uveitic syndromes with choroidal pathology, systemic uveitic syndromes with choroidal pathology, and multifocal choroiditis with outer retinal/choriocapillaris pathology with uveitis and without uveitis. Neoplastic and infectious etiologies are also discussed given their ability to masquerade as WDS.

## Background

The white dot syndromes (WDS) are a group of inflammatory disorders that affect the outer retinal layers, retinal pigment epithelium (RPE), and/or choroid [1]. WDS include: birdshot chorioretinopathy (BCR), multiple evanescent white dot syndrome (MEWDS), acute posterior multifocal placoid pigment epitheliopathy (APMPPE), multifocal choroiditis with panuveitis (MCP), serpiginous choroiditis (SC), punctate inner choroidopathy/multifocal choroiditis (PIC/MFC), and relentless placoid chorioretinitis (RPC). Although overlap and spectrums exist among these diseases, expanded imaging techniques in recent years have further clarified that each WDS represents a unique entity. More precise anatomic localization of the lesions has become possible with combined imaging modalities using spectral-domain optical coherence tomography (SD-OCT), enhanced depth SD-OCT imaging, fluorescein angiography (FA), indocyanine green angiography (ICGA), ultrasonography, wife-field imaging, and autofluorescence. In addition, OCT Angiography is a new modality that has been proven useful in the diagnosis of choroidal neovascularization (CNV). Therefore, anatomic classification of these diseases may be more appropriate in the current era, rather than lumping them as “white dot syndromes” (Table 1).

**Table 1:** Updated classification system

Uveitic syndromes with choroidal-based pathology
Sympathetic ophthalmia
Systemic uveitic syndromes with choroidal-based pathology
Vogt–Koyanagi–Harada disease
Sarcoidosis
Multifocal choroiditis with outer retinal/choriocapillaris-based pathology
Without vitritis
Histoplasmosis
Punctate inner choroidopathy
With vitritis
Multifocal choroiditis with panuveitis
Multiple evanescent white dot syndrome
Acute posterior multifocal placoid pigment epitheliopathy
Serpiginous choroiditis
Relentless placoid chorioretinitis
Birdshot chorioretinopathy
Neoplastic
Primary uveal lymphoma
Primary vitreoretinal lymphoma
Secondary (metastatic) Lymphoma
Infectious
Syphilis
Tuberculosis
Lyme disease

Treatment is not a focus of this review paper but it should be noted that in all of the following syndromes that anterior inflammation should be controlled with topical steroids and cycloplegics. Management of posterior sequelae is noted below where pertinent.

## Main text

### Uveitic syndromes with choroidal-based pathology

#### Sympathetic ophthalmia

Sympathetic ophthalmia (SO) is a bilateral granulomatous uveitis that occurs weeks to several decades following a penetrating injury or surgical trauma to an eye. Typically, the inflammatory process is confined to the choroid [2–4]. Early on, bilateral anterior cell and mutton-fat keratic precipitates can be observed. Thickening of the iris secondary to infiltration of inflammatory cells and posterior synechiae may also be seen. Posterior-segment examination may reveal vitritis, an exudative retinal detachment, and optic disc edema. The classic fundus finding is the presence of Dalen-Fuchs nodules which appear as focal, elevated yellowish-white lesions between the retinal pigment epithelium (RPE) and Bruch’s membrane [2, 3]. With disease progression, patients develop a “sunset glow fundus” secondary to depigmentation of the choroid.

Ultrasound reveals choroidal thickening and may disclose an exudative retinal detachment. Diffuse choroidal thickening, subretinal fluid, and irregular inner and outer segment (IS/OS) junction and external limiting membrane bands can be seen on SD-OCT [5, 6]. Fluorescein angiography demonstrates disk leakage and numerous progressively hyperfluorescent dots at the level of the RPE corresponding to pinpoint leakage. Occasionally, early focal blockage of the background choroidal fluorescence is seen [7]. Choroidal granulomas can be appreciated on ICGA as numerous hypocyanescent patches in the intermediate phase that may progress to isocyanescent in the late phase [8–10].

The prognosis for patients with SO dramatically improves with the use of corticosteroids and/or immunosuppressive agents [11, 12]. It is essential that treatment be initiated early in the course of the disease in order to prevent significant vision loss.

### Systemic uveitic syndromes with choroidal-based pathology

#### Vogt–Koyanagi–Harada disease

Vogt–Koyanagi–Harada (VKH) is a bilateral granulomatous uveitis. It is often associated with an exudative retinal detachment and extraocular manifestations, such as pleocytosis of cerebrospinal fluid, tinnitus, hearing loss, dysacusis, and cutaneous changes (e.g.: alopecia, poliosis, and vitiligo). It has a predilection for pigmented races such as Asians, Hispanics, American Indians, and Asian Indians [13, 14].

Patients may initially complain of a non-specific viral-like illness, including fever, nausea, headaches, dizziness, orbital pain, photophobia and meningism. Following this prodromal stage, patients will experience bilateral blurring of vision secondary to posterior uveitis. As the disease progresses the inflammation extends into the anterior segment leading to anterior chamber cell and flare including mutton-fat keratic precipitates. A few months after this uveitic phase, choroidal depigmentation leading to a sunset glow fundus is seen. Chorioretinal atrophy may also be apparent, with a predilection for the inferior mid-periphery of the fundus.

**Fig. 1:**
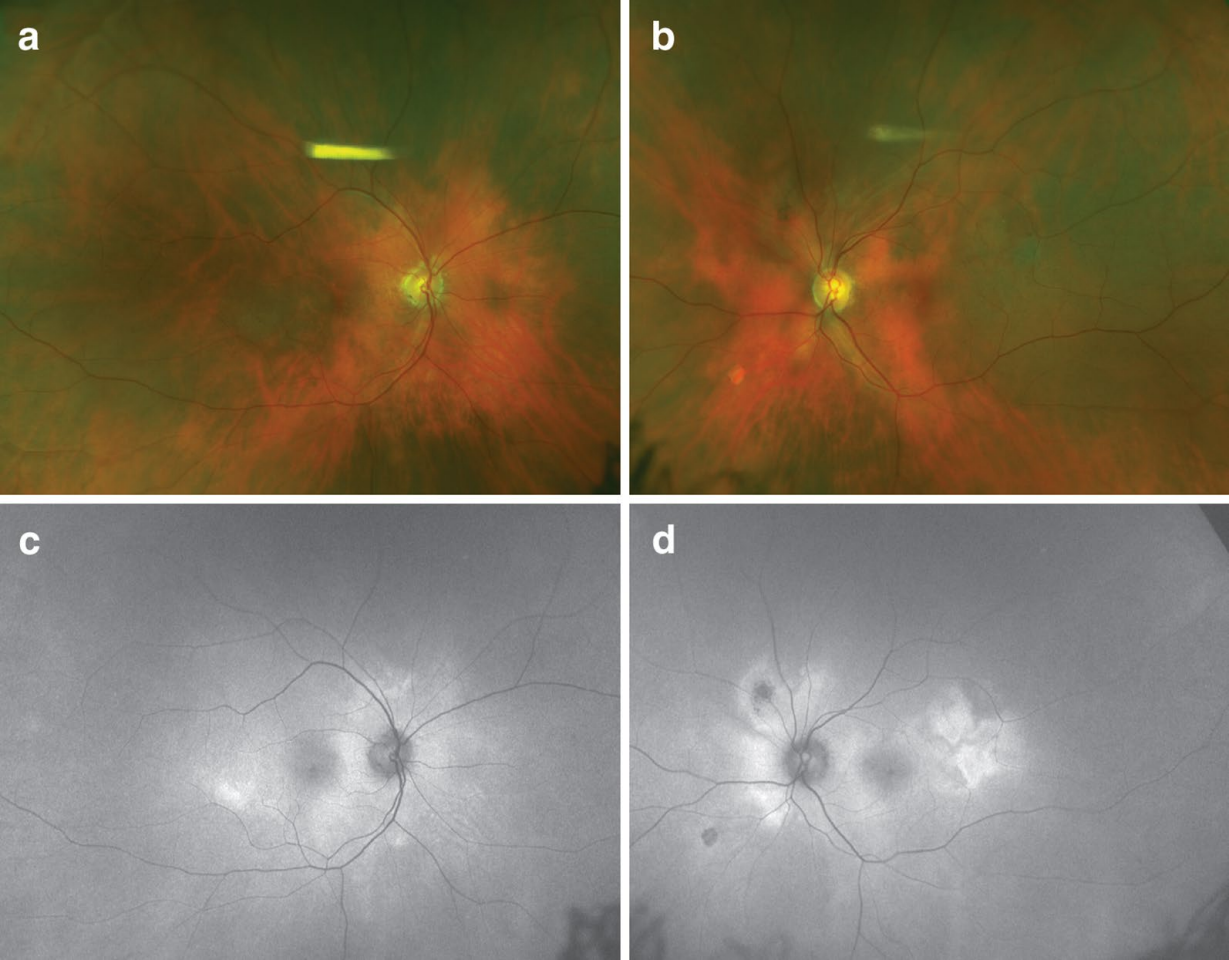
Sunset glow fundus in Vogt–Koyanagi–Harada (VKH) disease. Progressive choroidal depigmentation occurs after chronic VKH. Note the peripapillary depigmentation that gives the fundus a red appearance (**a**–**b**). Fundus autofluorescence shows patchy hyperautofluorescence (**c**–**d**)

Imaging studies are vital to diagnose and monitor the disease (Fig. 1). Ultrasound may reveal a shallow anterior chamber, cilio-choroidal detachment, thickened ciliary body, and serous retinal detachment. Enhanced depth imaging (EDI) OCT reveals a thickened choroid and can be used to monitor serous retinal detachments. Hypofluorescent pinpoint dots at the early phase of FA followed by multiple focal areas of leakage and subretinal dye accumulation at the late phase can be seen [15–17]. In chronic VKH, peripheral FAF abnormalities are seen. ICGA reveals early choroidal stromal vessel hypercyanescence and vascular leakage, and hypocyanescent dark dots at the level of the choroid in the late phase. Disc hyperfluorescence may also be seen [18, 19].

Treatment involves an extended course of corticosteroids, often greater than 6 months. The addition of immunomodulatory therapy should be utilized in uncontrolled cases. Better visual outcomes have been seen in cases with earlier treatment [20].

#### Sarcoidosis

Sarcoidosis is a granulomatous disorder of unknown etiology. The disease is multi-systemic with ocular involvement being found in 15–30% of sarcoid patients [21–25]. Although the disease can affect all races and genders, it is most frequently seen in African Americans [25].

Sarcoidosis can affect the orbit, adnexa, anterior and/or posterior segment. Anterior segment findings may include anterior uveitis with stereotypical mutton-fat keratic precipitates, iris nodules, conjunctival nodules and scleral nodules. Up to 60% of patients with ocular disease have posterior segment findings including vitritis, chorioretinitis, vascular occlusion, perivascular sheathing, neovascularization, and optic nerve head granulomas. Vitreous inflammation may clump to form “string of pearls” or “snowball” vitreous opacities. Periphlebitis is commonly found in the peripheral or mid-peripheral retina with severe periphlebitis being described as appearing as “candle-wax drippings.” Choroidal granulomas appear as isolated or multifocal elevated, subretinal, round-shaped, yellowish lesions. Large granulomas can have overlying serous retinal detachments. On EDI-OCT, Choroidal granulomas appeared as hyporeflective thickening of the choroid [26–28]. On FA, the lesions can demonstrate hypofluorescence, isofluorescence, early blocking with late staining, and hyperfluorescence [26, 29, 30]. Many reports have described the choroidal granulomas as hypofluorescent on ICGA [28, 31].

Treatment includes topical corticosteroid drops for anterior chamber inflammation and cystoid macular edema (CME). However, inflammation of the posterior segment typically involves the use of sub-Tenon’s corticosteroid injections, intravitreal triamcinolone acetonide, corticosteroid implant, or immunomodulatory therapy. Cycloplegic eye drops can be given to relieve ciliary spasm and to prevent the formation of posterior synechiae [25].

### Multifocal choroiditis with outer retinal/choriocapillaris-based pathology without vitritis

#### Ocular histoplasmosis syndrome

Ocular histoplasmosis syndrome (OHS) is a chorioretinal disorder due to an infection from Histoplasmosis capsulatum, a dimorphic fungus that is endemic to Mississippi and Ohio River valleys in the United States [32, 33]. Humans inhale infectious spores or conidia that subsequently disseminate into the bloodstream and eventually subside. After initial exposure to the fungus, patients may develop mild flu-like symptoms and asymptomatic calcified pulmonary nodules. Patients typically complain of visual symptoms including vision loss, metamorphopsia, and paracentral scotomata years following the resolution of the systemic infection when choroidal neovascularization (CNV) develops [34].

Ophthalmoscopy reveals an absence of vitritis or anterior segment inflammation. Posterior segment examination uncovers the classic triad of “punched-out” chorioretinal lesions in the mid-periphery and posterior pole (“histo spots”), chorioretinal peripapillary atrophy (PPA) and CNV. A general consensus is that at least two of the three posterior segment findings must be present in order to make the diagnosis of OHS. In the acute phase of disease, the histo spots will appear creamy-white and become slightly larger in size and more pigmented as the disease progresses. On OCT, in areas of affected outer retina (histo spots), there is loss of the intrinsic reflectance leading to the appearance of disorganization of the normal hyper-reflective bands [34]. In asymptomatic patients, FA findings show an early window defect pattern of hyperfluorescence with late progressive staining of the mid-peripheral atrophic spots and atrophic macular scars. If subretinal fluid or subretinal hemorrhage is present, early hyperfluorescence and late leakage from small blood vessels in the subretinal or subretinal pigment epithelial space is diagnostic of CNV. CNV can also be seen on OCT angiography (OCT-A). On fundus autofluorescence (FAF), OHS lesions correspond to areas of round hypoautofluorescence. ICGA may be helpful in evaluation of occult CNV exhibiting early increased hypercyanescence corresponding to new, disorganized choriocapillaris.

#### Punctate inner choroidopathy

**Fig. 2:**
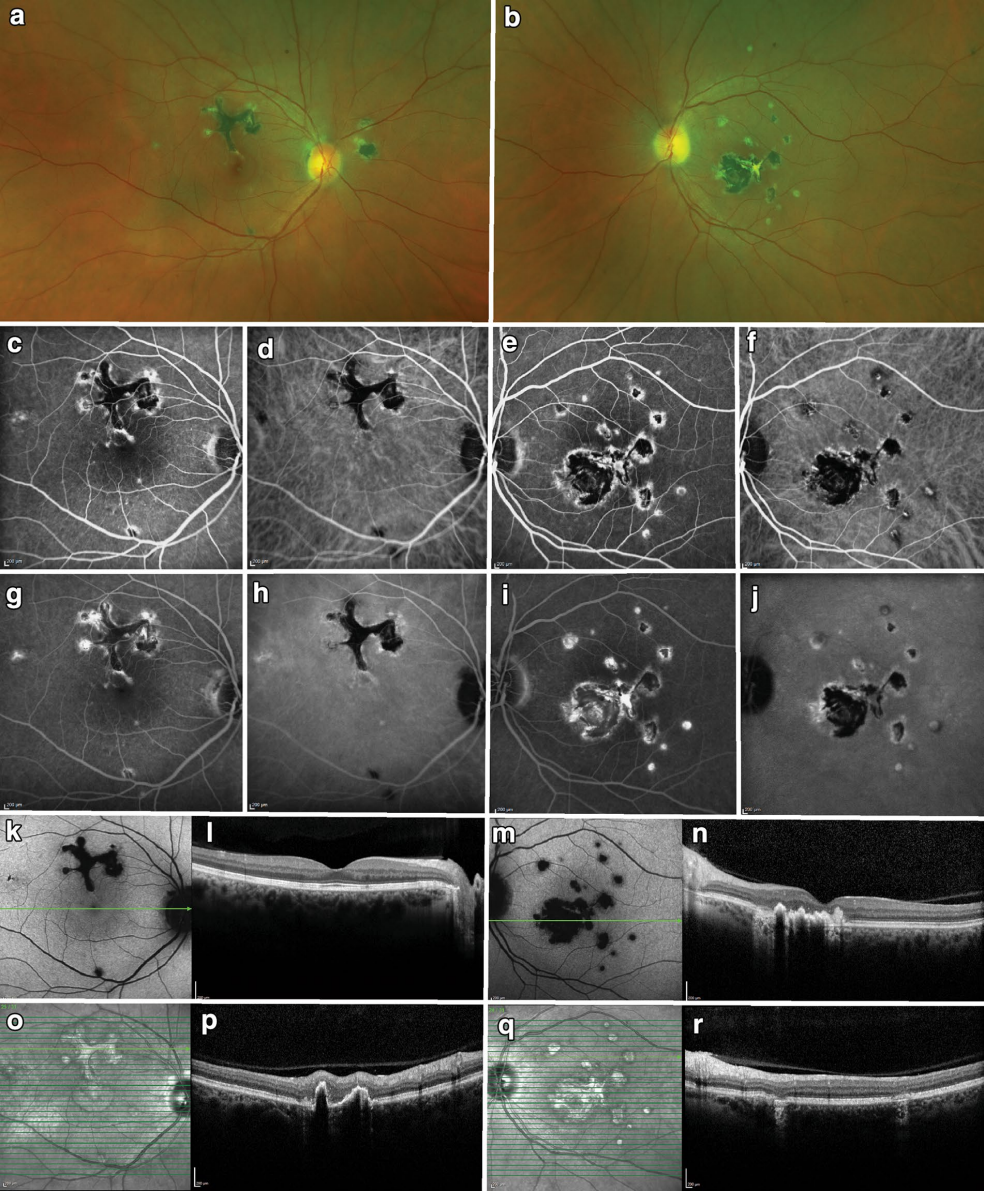
Punctate inner choroidopathy (PIC). A 43-year-old myopic woman presented with bilateral multifocal lesions (**a**–**b**). Fluorescein angiography (FA) showed blockage and mild rims of hyperfluorescence in earlier frames (**c**, **e**), which stained over time (**g**, **i**). Indocyanine green (ICG) showed similar patterns but more hypocyanescent overall (**d**, **h**, **f**, **j**). Fundus autofluorescence demonstrated hypoautofluorescence of the lesions seen on FA and ICG (**k**, **m**). Infrared imaging showed hyperreflectance of the lesions (**o**, **q**). Spectral-domain optical coherence tomography through the fovea of the right eye was normal (**l**), but the B-scans through the lesion superior to the fovea revealed disruption of the outer retina and retinal pigment epithelium (**p**). Similar lesions were noted in the *left eye* (**n**, **r**)

Punctate inner choroidopathy (PIC) tends to occur in Caucasian, young, myopic female (90%) patients. Patients complain of photopsias, blurred vision, paracentral scotomas, and metamorphopsia. Fundoscopic examination during the acute phase of disease shows discrete, small (100-300 μm), well-delineated, yellow-white lesions in the posterior pole at the level of the RPE, inner choroid or the choriocapillaris (Fig. 2) [35–37]. These lesions may coalesce and develop a serous retinal detachment. Additionally, they may gradually atrophy and become yellow-white chorioretinal scars, which may become pigmented over time. Mild optic disc edema can also be present. CNV is seen on initial presentation in 40–76% of patients [36, 38–40]. Examination does not show signs of anterior uveitis or vitritis [38].

OCT may reveal focal elevation of the RPE with underlying hyporeflective space and focal atrophy of the outer retina and RPE. Intraretinal fluid will be seen on OCT if CNV is present [41]. OCT angiography will detect CNV. The choroidal morphology seen on OCT includes presence of focal hyperreflective dots in the inner choroid and focal thinning of the choroid adjacent to PIC lesions [37, 42]. FA reveals more lesions than seen on clinical exam that appear as early hyperfluorescent lesions, and stain late. Atrophic lesions appear as window defects [1]. Leakage of fluorescence may be seen in the subretinal space if a serous detachment is present and late staining if CNV is present [36]. FAF shows active lesions as hypoautofluorescent spots with a hyperautofluorescent margin [43]. On ICGA, the lesions will appear hypocyanescent and will equal to the number seen on FA. No changes in electroretinography (ERG) or electrooculogram (EOG) are present.

CNV and CME can cause significantly impaired vision over time. Aggressive treatment of CNV can be associated with the maintenance of 20/40 or better visual acuity [44]. Recurrence is common and can be seen in 33–66.7% of patients [44–46].

### Multifocal choroiditis with outer retinal/choriocapillaris-based pathology with vitritis

#### Multifocal choroiditis with Panuveitis

Multifocal choroiditis with panuveitis (MCP) is a chronic, bilateral disease that generally affects young, healthy individuals, especially myopic females between the third and fifth decade of life [47, 48]. Patients complain of blurred vision, floaters and/or scotoma. They may also experience photopsias. The choroid, RPE, and retina are primarily involved [49].

Patients develop periodic episodes of clinically evident anterior uveitis and/or vitreous inflammation, differentiating it from ocular histoplasmosis. Yellow-white chorioretinal inflammatory lesions are visible in the posterior pole and periphery [50]. The lesions eventually evolve into punched-out scars with pigmented borders. Both eyes are generally affected, however, lesions may appear asymmetric due to delayed development between the two eyes. During active disease, a hyperemic disc, retinal vasculitis and CME can also be seen [51]. The most frequent cause of severe visual loss in these patients is from the development of CME and/or macular and juxtapapillary CNV [39, 52].

OCT reveals the presence of drusen-like sub-RPE material, choroidal hyperreflectivity below the lesions, and overlying vitreous cells [53]. On FA, acute lesions exhibit early hypofluoresence with late hyperfluorescent staining. CME and CNV may also be seen. FAF reveals hypoautofluorescent lesions in the posterior pole and periphery. ICGA imaging shows hypocyanescent spots within the choroid in quantities greater than lesions seen on ophthalmoscopy. Multifocal ERG generally reveals diffuse loss of function [54]. A recent study has shown the utility of OCT-angiography in diagnosis and evaluating response to treatment of associated CNV [55].

Disease reoccurrence is common [56]. Many patients with MCP have a poor visual prognosis due to disciform macular scarring, atrophy, or chronic CME [49, 50]. Treatment relies on the use of topical or periocular corticosteroids with use of systemic immunosuppressives if warranted. Secondary CNV can be managed expectantly with anti-VEGF agents.

#### Multiple evanescent white dot syndrome

MEWDS is an acute, multifocal, mostly unilateral disease affecting young adults. It affects females more than males with a ratio of 5:1 [57]. These patients are typically healthy and in their second to fourth decades of life. Roughly one half of the patients affected by this disease state that they had a prodromal flu-like illness preceding their ocular complaints [58]. Patients complain of acute onset of blurred vision, shimmering photopsias, dyschromatopsia, temporal vision loss, and paracentral or temporal scotomas [59]. Visual acuity may vary from 20/20 to 20/400 and a relative afferent pupillary defect may be present. Visual field testing may show an enlarged blind spot. There will be no anterior chamber inflammation. However, a mild vitritis is observed. The lesions are typically ill-defined and yellowish-white in color. They are located at the level of the RPE or outer retina and found predominantly in the perimacular area and extend out to the mid-peripheral retina. These routinely resolve within weeks to months and reoccurrence is rare. Mild pigmentary changes may develop following their resolution. Classically, foveal granularity is observed [60].

**Fig. 3:**
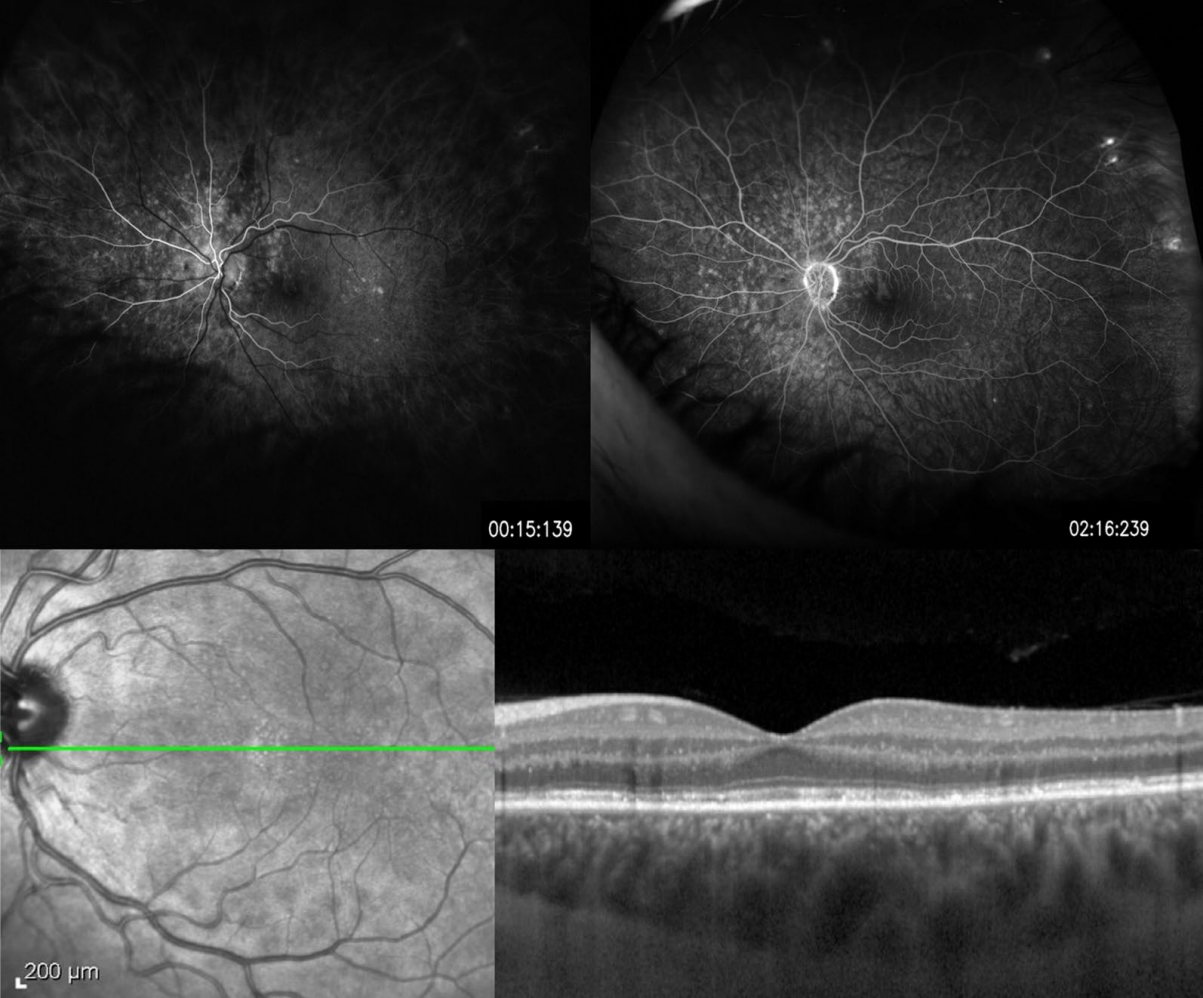
Multiple evanescent white dot syndrome. A 26-year-old woman presented with mildly decreased vision and photopsias. Fluorescein angiography revealed early hyperfluorescent lesions in a wreathlike configuration (*top left*) which persist into the late phase (*top right*). Optical coherence tomography shows disruption of the ellipsoid zone, and accumulation of hyperreflective material that rests on the RPE and extends anteriorly through the interdigitation zone, ellipsoid zone, and outer nuclear layer (*bottom right*)

OCT shows disruption of the ellipsoid zone (Fig. 3). Accumulation of hyperreflective material that rests on the RPE and extends anteriorly through the interdigitation zone, ellipsoid zone, and outer nuclear layer (ONL) can also be seen on OCT [61]. FA exhibits a variable number of early hyperfluorescent lesions in a wreathlike configuration in the mid-retina which persists into the late phase of the FA [61]. ICGA shows early and mid-phase hypocyanescent dots and/or plaques that are greater in number than those evident on FA. FAF demonstrates areas of hyperautofluorescence in the acute phase of the disease. Pinpoint hypoautofluorescence corresponding to the foveal granularity may also be seen [62]. A recent study examining wide-field FAF showed that the lesions first arise in the posterior pole and spread to the periphery during the acute stage; the lesions then fade from the periphery in a centripetal manner [63]. ERG may demonstrate reduced amplitude of the a-wave. The EOG may also be abnormal. However, both the ERG and EOG tend to normalize following resolution of the disease [64].

MEWDS has an overall good prognosis as most patients’ vision and visual fields are restored to baseline in several weeks to months. However, although rare, some patient may have a persistent blind spot enlargement, photopsias, and dyschromatopsia. No treatment is typically needed as most cases resolve spontaneously and recurrence is rare [60].

#### Acute posterior multifocal placoid pigment epitheliopathy

Acute posterior multifocal placoid pigment epitheliopathy (APMPPE) is an acute-onset inflammatory disease that affects the choriocapillaris, RPE, and outer retina. It typically affects young females, often in the second and third decades of life [1]. This disease is found equally between men and women [50]. Seventy-five percent of patients have bilateral presentation with the second eye affected within a few days or weeks following the first. The etiology is unknown; however, Gass felt APMPPE is often preceded by a viral prodrome [65]. Patients complain of bilateral, sudden, painless vision loss.

Ophthalmic examination does not reveal anterior inflammation; however, mild to moderate vitreous cell may be seen. Numerous, yellow, creamy colored placoid lesions are seen in the posterior pole and are not seen anterior to the equator. The lesions are often in various stages of evolution. A central clearing will be seen as they begin to resolve roughly within 2–3 months and progressively become hypopigmented. OCT exhibits hyperreflectivity of the outer retinal layers in the early stages which is thought to reflect swelling of the outer retinal cells or presence of inflammatory cell infiltrates [66, 67]. As the lesions resolve, hyperreflectivity of the outer retinal layers decreases. Disruption of the IS/OS junction and outer retina, and RPE atrophy can persist [68–71]. FA reveals lesions that characteristically demonstrate early hypofluorescence that subsequently hyperfluorescence in the late venous phase. The lesions seen on FAF are hypoautofluorescent and appear later and are less numerous than APMPPE lesions seen clinically. In the acute phase of disease, ICGA reveals more numerous hypocyanescent lesions than those seen on ophthalmoscopy. Studies have revealed that in the acute phase of disease, a large delay in choroidal filling as well as extensive areas of choroidal vessel nonperfusion can be seen. Furthermore, recovery of the choroidal blood flow is evident during clinical resolution [72–74]. OCT angiography has demonstrated these hypoperfused areas corresponding to the changes seen on ICGA [75]. Deutman et al. studied EOG and ERG recordings in APMPPE patients. ERG findings revealed marginally abnormal values of the a- and b-wave amplitudes in the acute phase of disease. The EOG recordings were also abnormal in the acute phase but showed improvement with disease resolution [76] which is usually seen within 2–3 months. These course is often self-limiting and patients typically have a good visual prognosis. However, foveal involvement, older age, unilateral disease, and recurrent disease are features that may contribute to poor visual prognosis. In these cases systemic steroids have been reported as beneficial but further studies are needed to determine if there is true efficacy [59].

#### Serpiginous choroiditis

Serpiginous choroiditis (SC) is a rare condition that affects men slightly more than women in their second to seventh decades of life [50, 59, 77–79]. It typically is a bilateral disease that is chronic and progressive in nature. The RPE, choriocapillaris, and choroid are involved [80–83].

**Fig. 4:**
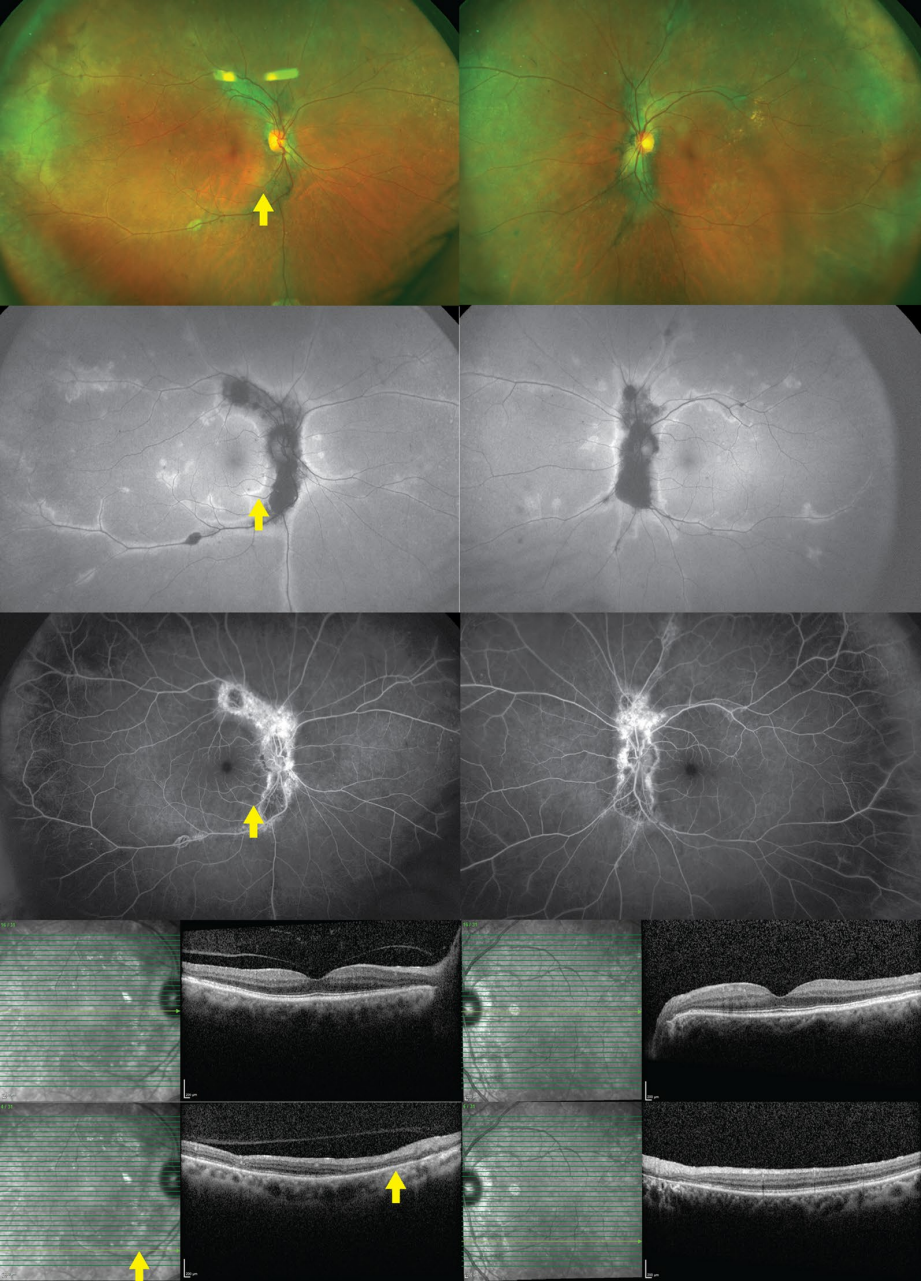
Serpiginous choroiditis. A 68-year-old woman presented with chronic visual field defects in both eyes. Lesions deep to the retina were noted bilaterally emanating from the optic disc (*top row*). Fundus autofluorescence revealed central hypoautofluorescence with rims of hyperautofluorescence (*second row*). Fluorescein angiography showed central patches of hypofluorescence with hyperfluorescent staining over time (*third row*). Central spectral-domain optical coherence tomography revealed a normal fovea, but there was disruption of the ellipsoid zone, external limiting membrane, and retinal pigment epithelium, nasally (*bottom rows*). The *yellow arrow* corresponds to the same area illustrated in all images

Although SC usually has bilateral involvement, patients typically present with unilateral decreased central vision, metamorphopsia, or scotoma. Anterior segment inflammation is usually mild, and the vitreous may be clear or show minimal inflammation. Ophthalmoscopy reveals lesions originating in the peripapillary region or macula in active disease that appear as areas of grayish or creamy yellow sub-retinal infiltrates. These lesions progress in an irregular serpentine or helicoid fashion centrifugally (Fig. 4). Overlying retina edema is also present. Different stages of healing are seen between the lesions as they resolve, typically around 8-weeks. This resolution leaves areas of atrophy involving both the choriocapillaris and RPE. Multiple recurrences are common and new lesions are classically found at the edge of previous atrophic scars. Macular involvement is associated with worse visual prognosis and higher risk of secondary CNV.

Active lesions on OCT show hyper-reflectivity and thickening of the outer retina. There is also increased reflectance of the choroid which has been referred to as the ‘waterfall’ effect. The photoreceptor inner and outer segment (IS/OS) junction in both active and inactive lesions will be disrupted [84]. FA of the active lesions show early hypofluorescence and late hyperfluorescence of the border in a typical geographic pattern. Old lesions show window defects, and late staining. ICGA can be divided into four stages. The first reveals hypocyanescent lesions in the subclinical or choroidal stage. The second, hypocyanescent lesions in the active phase. Third, hypercyanescence in the healing and sub-healing stage and the fourth is hypocyanescent lesions with clearly defined margins in the inactive phase [50, 85]. FAF shows new hyperautofluorescent lesions appearing at the edge of old lesions which are hypofluorescent [85]. Electrophysiologic studies are usually normal.

Rapid control of the active lesions with periocular and systemic corticosteroids are necessary to limit extensive scarring and secondary CNV [77, 87]. Long-term steroid-sparing therapy such as cyclosporine, azathioprine, cyclophosphamide, interferon alpha-2a, or mycophenolate mofetil may be needed to prevent recurrence [78, 88–91]. It should be noted that tuberculosis can cause a serpiginous-like choroidopathy (SLC). SLC is less likely to respond to sole treatment with systemic corticosteroids or immunosuppressants making Tb testing mandatory especially in endemic regions. Differentiating SC and SLC based on imaging findings is discussed in the infectious section below.

#### Relentless placoid chorioretinitis

Relentless placoid chorioretinitis (RPC) is a chronic, relapsing disease of unknown etiology. Men and women are equally affected typically in their second to sixth decades of life [92]. Patients may complain of blurred vision, metamorphopsia, pericentral scotomas, photopsias, and/or floaters [93]. It was formerly known as ampiginous chorioretinitis.

This disease is often confused with APMPPE and SC. RPC can appear very similar to APMPPE or macular serpiginous initially, and the relentless course beyond 6 months is often the key to its diagnosis and differentiation from APMPPE. Ophthalmoscopy reveals creamy white lesions roughly one-half disc diameters in size at the level of the RPE initially in the posterior pole [94]. The lesions are often found bilaterally affecting the mid- and far periphery first with subsequent involvement of the posterior pole and/or macula. The lesions can remain active and spread or heal causing pigmented chorioretinal atrophy. The presence of > 50 to hundreds of lesions in different stages of activity found anterior and posterior to the equator is characteristic of this disease entity [1]. Anterior chamber and vitreous cells are typically seen. A similar condition is persistent placoid maculopathy, which is more in the MEWDS spectrum. RPC can be distinguished from persistent placoid in that RPC tends to occupy the peripheral retina.

**Fig. 5:**
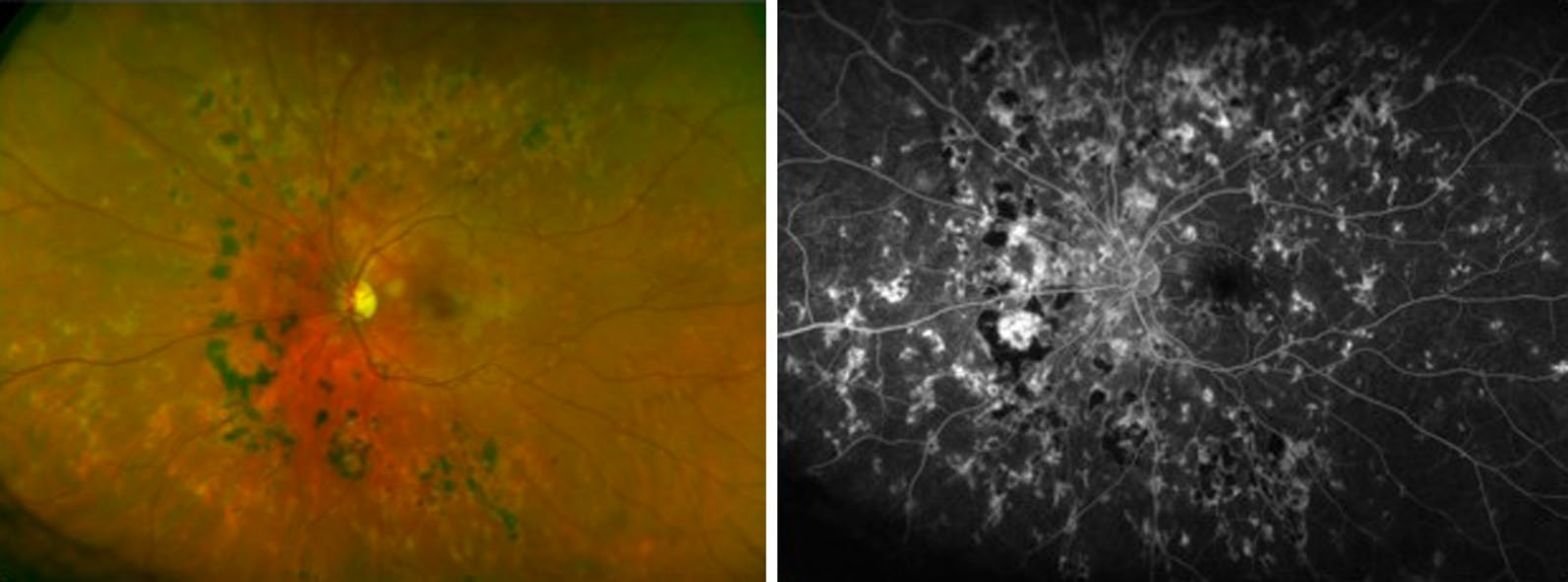
Relentless placoid chorioretinitis. Numerous chorioretinal scars of different ages are evident in the fundus photo (*left*) of this 25 year-old patient with relentless placoid chorioretinitis. Similar findings were present in the other eye. Fluorescein angiography (*right*) demonstrates staining of the chorioretinal scars in the late phase with blockage from the older hyperpigmented regions. These lesions were deemed inactive

If foveal lesions are present in RPC, OCT may reveal subfoveal fluid. In addition, a pigment epithelial detachment (PED) with hyperreflectivity of the inner and outer retinal layers can also be seen [95]. FA reveals early hypofluorescence and late staining (Fig. 5). Yeh et al. [86] looked at FAF imaging of a patient with RPC which displayed widespread hypoautofluorescence involving the posterior pole and mid-peripheral retina. Hypocyanescence in the areas corresponding to the clinical lesions that persists into the late phases is seen on ICGA [92, 93]. ERG and EOG are normal. Treatment involves systemic corticosteroids with addition of immunosupressives if warranted. Although this is a chronic disease and relapse is common, the long-term visual prognosis is generally good.

#### Birdshot chorioretinopathy

Birdshot chorioretinopathy (BCR) has a slight female predominance and is typically found in patients between the ages of 40 and 60 [96–98]. The etiology of BCR remains unclear, however studies have shown that of the patients who have BCR, nearly 90% possess human lymphocyte antigen (HLA) A29. This is the highest association of any HLA antigen with a human disease [97]. Testing for HLA-A29 in useful in making the diagnosis as it has a sensitivity of 96% and specificity of 93% [48, 99].

**Fig. 6:**
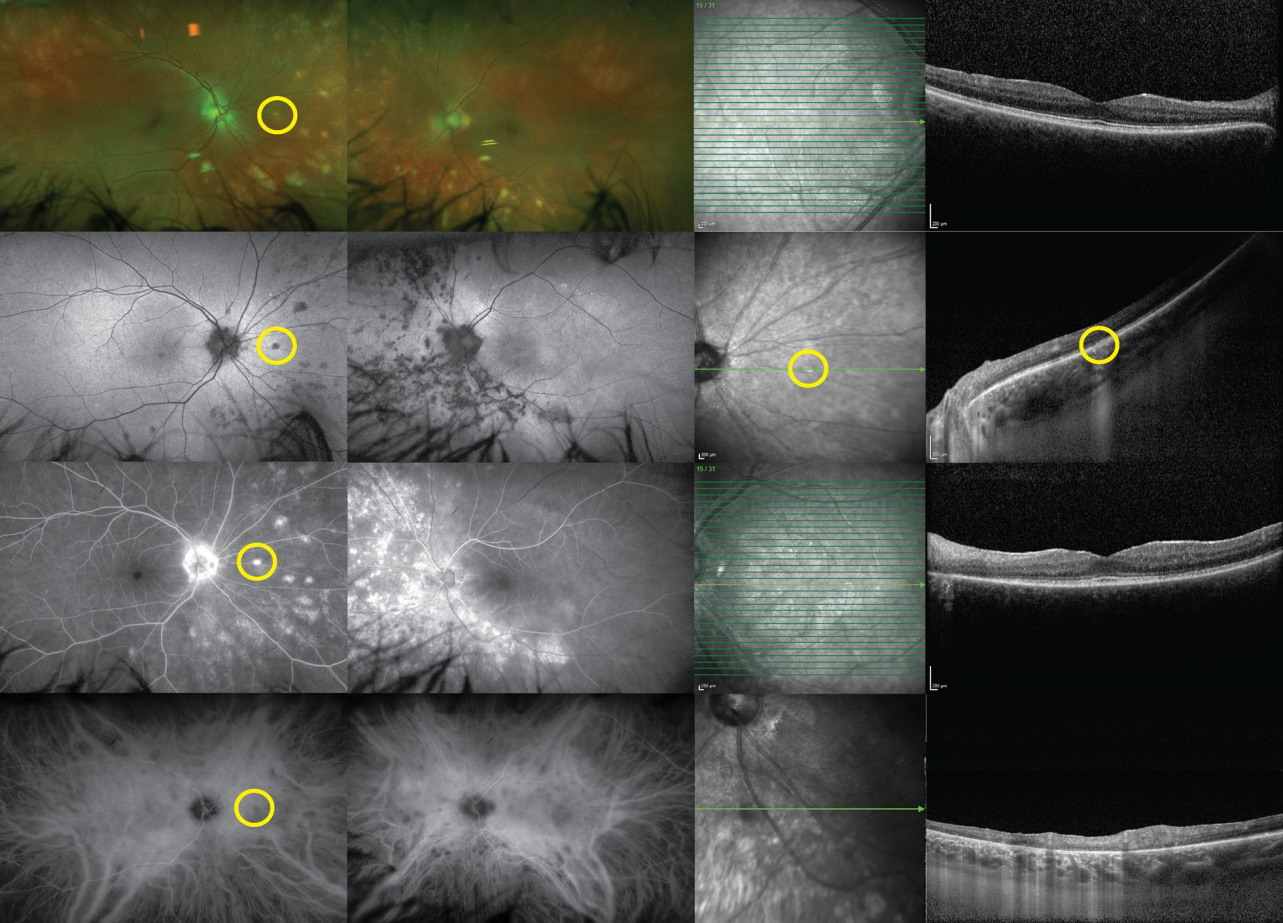
Birdshot uveitis. A 58-year-old woman presented with photophobia in both eyes. White, deep, multifocal, choroidal lesions were seen on examination, mostly located nasally (*top row*). Fundus autofluorescence showed that these lesions were hypoautofluorescent (*second row*). The lesions were hyperfluorescent on fluorescein angiography (*third row*), and corresponded to hypocyanescent spots on indocyanine *green* (*bottom row*). Spectral-domain optical coherence tomographies of the fovea in both eyes were relatively unremarkable. Enhanced-depth imaging of lesions seen on previous imaging (*yellow circles*) demonstrates focally disrupted outer retinal structures with increased transmission into the choroid. Work-up revealed HLA-A29 positivity

Patients may initially complain of blurred vision that is often out of proportion to the visual acuity loss, floaters, photopsia, and later may experience nyctalopia or color blindness. Creamy lesions seen on fundus examination are typically oval or round in shape, and one-quarter to one-half disc diameter in size. They can present asymmetrically between the eyes and be subtle in appearance. The lesions typically cluster around the optic disk, most commonly nasal and inferior to the disk, and radiate out to the equator (Fig. 6) [100]. Vitreous inflammation is seen in nearly every patient. However, inflammation in the anterior segment is generally absent. Retinal vasculitis manifests as narrowed retinal vessels [59]. CME, and disc edema can also be seen [96].

OCT is typically employed to follow CME. Interestingly, a recent study used extramacular EDI-OCT and found BCR patients had focal or generalized disruption of the photoreceptor IS/OS junction. Many patients exhibited thinning or absence of the Sattler layer, or had an appearance of generalized atrophy of the choroid [101]. FA reveals optic disc hyperfluorescence, vascular leakage, late CME, and prolonged arteriovenous transit time [97, 102]. This delayed transit time is a phenomenon call “quenching” and is unique to BCR [48]. With FAF, hypoautofluorescent areas corresponding to areas of chorioretinal atrophy are noted [86]. ICGA is a sensitive diagnostic test and useful at visualizing the birdshot lesions as areas of blockage in the early to midphase. A recent report showed a series of patients with BCR exhibiting lesions on ICGA before the lesions were visible on clinical examination or FA [103]. ERG has been shown to be abnormal in 88.8% of patients in a study that analyzed data from 10 published articles (89 patients) [97]. Classically delayed 30-Hz implicit time and diminished scotopic b-wave amplitudes will be seen [104]. The ERG is an important tool for studying progression of disease. EOG is typically normal. Visual field examinations are important as peripheral constriction, enlarged blind spot, central or paracentral scotomas can be present.

Long-term prognosis is guarded as the disease is chronic and does not appear to regress. The causes of vision loss is multifactorial and includes loss of photoreceptors, macular edema, and disc edema leading to optic disc atrophy. In the short term, corticosteroids can be used. However, due to their side effects, steroid-sparing medications like cyclosporine, azathioprine, mycophenolate mofetil, methotrexate or biologics should be considered for long-term management.

### Neoplastic

Three distinct forms of intraocular lymphoma exist and include primary vitreoretinal (PVRL), primary uveal and secondary (metastatic) lymphoma. Primary vitreoretinal lymphoma (PVRL) affects the vitreous and RPE. Uveal tissue is the primary site of involvement in metastatic and uveal lymphoma [105–107]. Most patients present with painless, decreased visual acuity or floaters [108, 109].

PVRL is the most common intraocular lymphoma and is often associated with central nervous system (CNS) disease. It is commonly seen in older or immunocompromised patients. Most cases are bilateral, but asymmetric in presentation [110, 111]. The presence of clumped vitreous cells and multiple irregular yellowish white sub-RPE deposits are pathognomonic features [112–115]. Punched-out lesions leading to a disciform-like scar, retinal vasculitis, solid RPE detachment or exudative retinal detachment can also be present. OCT can show pigment epithelial detachments and exudates above the RPE [116]. EDI-OCT is particularly useful for choroidal lymphoma that has thin tumor infiltration. It may show a “placid, rippled, or stormy (seasick)” appearance depending on the thickness of invasion [117]. FA findings can vary and may reveal staining of subretinal deposits, RPE window defects, and diffuse RPE granularity [106, 118]. FAF will show hyperautofluorescence of the RPE over the lymphoma deposits. Retinal deposits overlying the RPE are hypoautofluorescent due to blocking. A study by Casady et al. showed that the majority of patients have a granular hyperautofluorescence and hypoautofluorescence on FAF. All of these patients were also found to have active disease at the time of imaging [119].

Primary uveal and secondary (metastatic) lymphoma usually present unilaterally [120]. The majority of patients have vitreous cell on examination. Anterior cell and flare is rare. Characteristically, multiple yellow subretinal infiltrates will be seen on fundus examination that may result in overlying RPE detachments [121]. Creamy thickening of the choroid diffusely and RPE clumping may also be present. Collections of tumor cells in the sub-RPE can be seen on OCT. FA may demonstrate RPE granularity, blockage by RPE pigment clumps or disrupted RPE, and late staining [118, 122]. Tumor cell infiltrates may be seen as round hypofluorescent lesions [123]. Diffuse uveal thickening, subretinal masses, and intravitreal cells can be seen on ultrasonography. Typically, ICGA reveals hypocyanescent lesions corresponding to the clinically observed choroidal infiltrates [120].

### Infectious

Some infectious etiologies can be confused for the WDS’s, including syphilitic chorioretinitis, ocular tuberculosis (TB), and Lyme disease. These infections are treatable masquerade syndromes, thus any suspicion in the history or exam findings should prompt a laboratory workup.

**Fig. 7:**
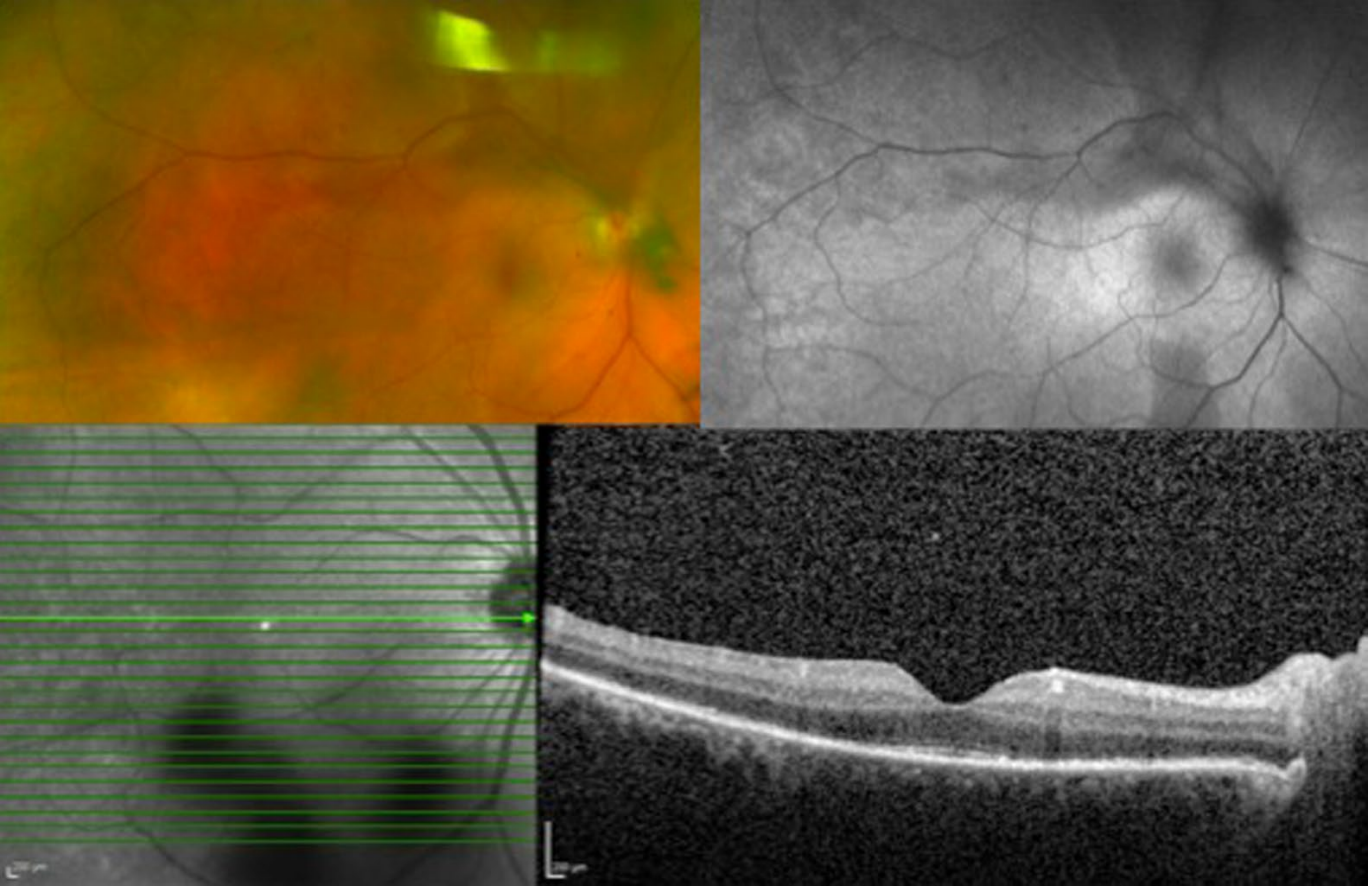
Syphilitic Uveitis. A 55 year-old man presented with new onset panuveitis in the *right eye*; he was found to be seropositive for syphilis. *Color* fundus photo (*top left*) and autofluorescence (*top right*) of the *right eye* demonstrate the characteristic placoid appearance of the syphilitic lesions. Optical coherence tomography (*bottom*) reveals segmental loss of ellipsoid zone

Syphilitic inflammatory lesions can be seen affecting the outer retina, RPE, and choroid. The lesions in the fundus may appear as yellow, placoid, chorioretinal lesions in the posterior pole or within the macula (Fig. 7) [124]. A faded center and clumping of the adjacent retinal pigment epithelium may be present. OCT may reveal areas of loss in the IS/OS junction and external limiting membrane [125, 126]. Hypofluorescent areas can be seen on FA corresponding to the fundus lesions. Sporadically leopard spotting may be seen as hypo- and hyperfluorescence in the faded part of the lesions, followed by progressive hyperfluorescence [127]. ERG may be markedly reduced [128].

**Fig. 8:**
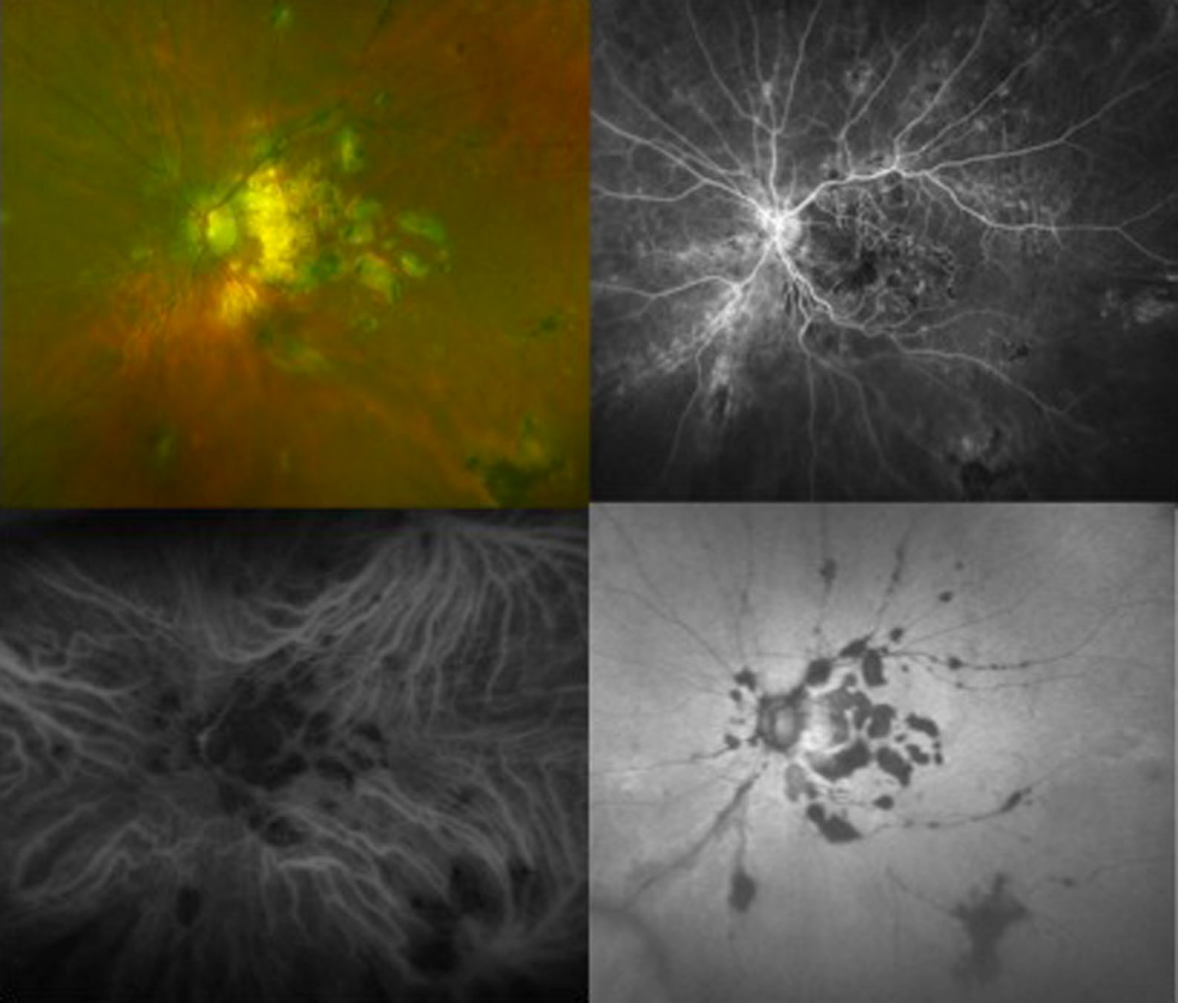
Panuveitis Secondary to Tuberculosis. *Color* fundus photograph (*top left*) of a 50 year-old woman with a history of tuberculosis developed serpiginous choroidal atrophy in the *left * and *right eyes*. Fluorescein angiography (*top right*), indocyanine *green* angiography (*bottom left*) and fundus autofluorescence (*bottom right*) demonstrate areas of hypofluorescence corresponding to the regions of chorioretinal atrophy

Ocular tuberculosis (TB) most commonly affects the choroid. Choroidal tuberculomas, choroidal tubercles, multifocal choroiditis, and serpiginous-like choroiditis can be seen [129, 130]. Miliary choroidal tubercles appear as small flat, yellow-white lesions with indistinct borders in the choroid and can grow into a single mass lesion called a tuberculoma (Fig. 8). A study by Salman et al. studied OCT findings in nine patients with choroidal tuberculosis and found areas of localized adhesion between the RPE-choriocapillaris layer and the overlying neurosensory retina. They termed this the “contact sign” [131]. FA of choroidal tubercles show hypofluorescent lesions during dye transit then become hyperfluorescent in the late frames. However, healed tubercles will show transmission hyperfluorescence. Choroidal tuberculomas show early hyperfluorescence that increases in hyperfluorescence, with pooling of dye during the late phase secondary to an exudative retinal detachment [132]. FA in serpiginous-like choroiditis reveals hypofluorescence of the active edge initially with late hyperfluorescence of the advancing edge [133]. ICGA of choroidal lesions in the acute phase will appear hypofluorescent during the initial and transit period. However, as the angiogram progresses into the late phase, the edges become hyperfluorescent. In contrast, active lesions seen in serpiginous-like choroiditis are seen as hypofluorescent spots during the early and late phases of ICGA. Patients with ocular Lyme disease characteristically complain of photophobia and severe periodic ocular pain [134]. Choroiditis has been reported [134, 135] and described as multiple, well circumscribed creamy lesions seen deep in the retina with pigmentation and choroidal atrophy. FA in one case revealed deep hypofluorescence of the creamy lesions that contrasted with the granular hyperfluorescence of the lesions seen in the periphery.

## Conclusion

Given their overlapping historical, clinical and imaging features, the white dot syndromes and their masqueraders represent a difficult diagnostic challenge for many clinicians. Distinguishing these entities is important as some of these entities such as APMPPE have a self-limited course and an excellent visual prognosis while others such as birdshot chorioretinopathy have a chronic course and a more guarded prognosis. Furthermore, accurate diagnosis can ensure appropriate monitoring is initiated. Entities such as PIC must be monitored closely to detect development of CNV to ensure early vision saving treatment. Correct diagnosis can also aid in laboratory testing for associated autoimmune conditions or infectious associations. Ongoing research and expanded imaging techniques have made it apparent that each syndrome represents a unique entity with specific anatomic localization (Table 2). Combining imaging modalities including SD-OCT, enhanced depth SD-OCT, FA, ICGA, ultrasonography, wide-field imaging, autofluorescence, and now OCT angiography can greatly help in distinguishing between the various WDS’s.

**Table 2:** Clinical and multimodal imaging findings seen in the white dot syndromes and their masqueraders

	Sympathetic ophthalmia	VKH	Sarcoidosis	OH	PIC	MCP	MEWDS	APMPPE
Anterior exam findings	Possible AC cell and mutton-fat keratic precipitates; thickened iris	AC cell and flare	AC cell with mutton-fat keratic precipitates; Iris, conunctival and/or scleral nodules	No inflammation	Typically absent inflammation	Periodic AC cell and flare	No inflammation	No AC inflammation
Posterior examination findings	Vitritis; depigmentation of the choroid; Dalen-Fuchs nodules	Choroidal depigmentation; Possible chorioretinal atrophy	“String of pearls” or “snowball” vitreous opacities; peripheral periphlebitis; choroidal granulomas; optic nerve head granulomas	“Punched-out” chorioretinal lesions in the mid-periphery and posterior pole (“histo spots”); chorioretinal peripapillary atrophy (PPA); CNV	Small (100–300 μm), well-delineated, yellow-white lesions in posterior pole at the RPE, inner choroid or choriocapillaris; Possible yellow-white chorioretinal scars; CNV	Greyish-yellow, jogsaw-puzzle-shaped lesions at levels of the RPE and choriocapillaris emanate from optic nerve; lesions evolve into punched-out scars with pigmented borders; CNV	Ill-defined round, yellowish–white lesions in perimacular area and occasionally peripheral to the arcades; granular fovea	Vitritis; numerous, yellow, creamy colored placoid lesions are seen in posterior pole in various stages of evolution
OCT	Diffuse choroidal thickening, subretinal fluid, and irregular IS/OS junction and ELM	Thickened choroid; possible sereous retinal detachment	Hyporeflective thickening of the choroid	Loss of intrinsic reflectance	Focal elevation of RPE with underlying hyporeflective space and focal atrophy of the outer retina and RPE; focal hyperreflective dots in inner choroid; focal thinning of choroid adjacent to lesions	Drusen-like sub-RPE material; choroidal hyperreflectivity below lesions, and overlying vitreous cells	Disruption of ellipsoid zone; accumulation of hyperreflective material that rests on RPE and extends anteriorly	Hyperreflectivity of outer retinal layers in early stages; disruption of IS/OS junction and outer retina; RPE atrophy
Fluorescein autofluorescence		Peripheral FAF abnormalities		Lesions correspond to round hypoautofluorescence	Hypoautofluorescent spots with hyperautofluorescent margin	Hypoautofluorescent lesions in pole and periphery	Areas of hyperautofluorescence in acute phase; possible pinpoint hypoautofluorescence corresponding to foveal granularity	Hypoautofluorescent lesions that appear later and less numerous than APMPPE lesions seen clinically
Fluorescein angiography	Disk leakage; numerous progressively hyperfluorescent dots at level of the RPE; possible early focal blockage of background choroidal fluorescence	Hypofluorescent pinpoint dots in early phase followed by multiple focal areas of leakage and subretinal dye accumulation at late phase	Hypofluorescence, isofluorescence, early blocking with late staining, and hyperfluorescence	Early window defect pattern of hyperfluorescence with late progressive staining of mid-peripheral atrophic spots and atrophic macular scars	Early hyperfluorescence, late staining (more than seen on exam); window defects of atrophic lesions	Early hypofluoresence with late hyperfluorescent staining	Early hyperfluorescent lesions in wreathlike configuration in mid-retina	Early hypofluorescence that subsequently hyperfluorescence in late venous phase
ICGA	Numerous hypocyanescent patches in intermediate phase that progress to isocyanescent in late phase	Early choroidal stromal vessel hypercyanescence and vascular leakage; hypocyanescent dark dots at level of choroid in late phase; possible disc hyperfluorescence	Hypofluorescent granulomas	Early increased hypercyanescence from CNV	Hypofluorsecent spots (same number as seen on FA)	Hypocyanescent spots within choroid (quantities greater than lesions seen on exam)	Hypocyanescent dots in early to mid-phases	More numerous hypocyanescent lesions than those seen on ophthalmoscopy
ERG/EOG	Normal	Normal	Normal	Normal	Normal	Diffuse loss of function	ERG: reduced a-wave; ± abnormal EOG; both typically normalize following resolution	ERG: moderate reduction of a- and b-wave amplitudes in acute phase; EOG: abnormal in acute phase but improves with disease resolution

	SPC	RPC	BCR	Neoplastic-PVRL	Choroidal lymphoma	Syphilis	Tuberculosis
Anterior exam findings	Mild AC inflammation	AC inflammation	Typically absent AC inflammation	Rare anterior cell	AC inflammation is less common	AC inflammation common	AC inflammation
Posterior examination findings	Vitritis; grayish or creamy yellow sub-retinal infiltrates in peripapillary region or macula that progress in irregular serpentine or helicoid fashion centrifugally	Vitirits; numerous creamy white lesions initially in peripherally then involvement of posterior pole or macula; bilateral	Vitirits; multiple cream or yellowish-white oval lesions varying in size from 1/4 to 1 disk diameter; longer diameter radiating from optic nerve to the periphery	Clumped vitreous cells and multiple irregular yellowish white sub-RPE; punched-out lesions leading to a disciform-like scar; retinal vasculitis	Vitreous cell; multiple yellow subretinal infiltrates; creamy thickening of choroid diffusely and RPE clumping	Yellow, placoid, chorioretinal lesions in posterior pole or within macula	Small flat, yellow-white lesions with indistinct borders in the choroid
OCT	Hyper-reflectivity and thickening of outer retina; increased reflectance of choroid; disruption of IS/OS junction	Pigment epithelial detachment with hyperreflectivity of inner and outer retinal layers	CME; focal or generalized disruption of IS/OS junction; possible thinning or absence of Sattler layer; possible appearance of generalized atrophy of the choroid	“Placid, rippled, or stormy (seasick)” appearance possible pigment epithelial detachments and exudates above the RPE	Tumor cells in sub-RPE	Loss of IS/OS junction and ELM	“Contact sign”—localized adhesion between RPE-choriocapillaris layer and overlying neurosensory retina
Fluorescein autofluorescence	Active lesions are hyperautofluorescent; inactive lesions are hypoautofluorescent	Widespread hypoautofluorescence involving the posterior pole and mid-peripheral retina	Hypoautofluorescent areas corresponding to areas of chorioretinal atrophy	Hyperautofluorescence of RPE over lymphoma deposits; hypoautofluorescent retinal deposits overlying the RPE			
Fluorescein angiography	Early hypofluorescence and late hyperfluorescence of the border; Window defects of old lesions	Early hypofluorescence and late staining	Optic disc hyperfluorescence; vascular leakage; late CME; prolonged arteriovenous transit time (“quenching”)	Staining of subretinal deposits; RPE window defects; diffuse RPE granularity	RPE granularity; blockage by RPE pigment clumps or disrupted RPE; late staining	Hypofluorescent lesions; hypo- and hyperfluorescence in faded part of the lesions, followed by progressive hyperfluorescence	Active tubercles—hypofluorescent lesions during dye transit then hyperfluorescent in late frames; tuberculomas—early hyperfluorescence; serpiginous-like choroiditis—hypofluorescence of active edge with late hyperfluorescence of advancing edge
ICGA	Hypocyanescent lesions during active phase; hypercyanescence in healing phase; hypocyanescent lesions with clearly defined margins in inactive phase	Hypocyanescence lesions that perist into late phase	Areas of blockage in early to midphase		Hypocyanescent lesions		
ERG/EOG	Normal	Normal	ERG: delayed 30-Hz implicit time and diminished scotopic b-wave amplitudes; normal EOG	Normal		ERG: possibly markedly reduced. EOG: normal	

## Abbreviations

WDS: white dot syndromes
BC: birdshot chorioretinopathy
MEWDS: multiple evanescent white dot syndrome
APMPPE: acute posterior multifocal placoid pigment epitheliopathy
MCP: multifocal choroiditis
SC: serpiginous choroiditis
RPC: relentless placoid chorioretinitis
PIC: punctate inner choroidopathy
SO: sympathetic ophthalmia
VKH: Vogt–Koyanagi–Harada disease
OHS: ocular histoplasmosis syndrome
PVRL: primary vitreoretinal
CNS: central nervous system
TB: tuberculosis
EDI: enhanced depth imaging
SD-OCT: spectral-domain optical coherence tomography
FA: fluorescein angiography
FAF: fundus autofluorescence
ICGA: indocyanine green angiography
RPE: retinal pigment epithelium
IS/OS: inner and outer segment
CME: cystoid macular edema
CNV: choroidal neovascularization
PPA: peripapillary atrophy
ONL: outer nuclear layer
ERG: electroretinography
EOG: electrooculogram
